# “Few things in life are easy *and* worth doing”: how the bi-directional relationships between meaningful work and work-related stress can both help and hinder wellbeing

**DOI:** 10.3389/fpsyg.2023.1244051

**Published:** 2023-10-30

**Authors:** Jess Annison, Adam Davidson

**Affiliations:** School of Psychology, University of East London, London, United Kingdom

**Keywords:** meaningful work, work-related stress, wellbeing, job demands-resources model, employee engagement

## Abstract

**Introduction:**

Meaning is a key part of psychological wellbeing, and the benefits of meaningful work are widely acknowledged. Many people seek meaning from their work, and some organizations aim to facilitate this through interventions. In parallel, work-related stress has become a significant occupational risk. This study seeks to understand the perspectives of those who find their work to be both meaningful and stressful, and to explore the relationships between these concepts.

**Methods:**

Eleven women and six men, aged 34–61, primarily based in the UK, from the private, public, and third sectors were interviewed about their experiences of meaning and stress in their work. Using a social constructivist grounded theory approach, data collection and analysis ran in parallel.

**Results:**

Findings indicate that meaningful work and work-related stress are inherently connected, with bi-directional relationships that can support and hinder wellbeing. Meaningfulness can both alleviate and exacerbate stress, and stress can both reinforce and reduce meaningfulness. Meaningfulness and stress can even feel co-dependent, depending on how participants perceive and make sense of their experience.

**Discussion:**

With many individuals seeking greater meaningfulness from their work, the results suggest that they— and their employers—would benefit from understanding more about the potential harmful effects of meaningfulness, including implications for stress and possible knock-on consequences for health and work.

## Introduction

Work-related stress is a significant occupational risk within industrialized countries (Hassard et al., 2018). Stress is experienced when an individual faces challenging circumstances that, in their judgment, exceed their resources and ability to cope (Lazarus and Folkman, 1984). Other definitions refer to stress as either a cause or effect (or both), making stress challenging to analyze (Johnston, 1995). When stress is experienced moderately and sporadically, it is beneficial for our growth and development (Brulé and Morgan, 2018). However, when stress becomes acute or chronic, it can cause problematic physiological and psychological effects (Cooper et al., 2001) such as cardiovascular disease, reduced immune functioning and depression (Michie, 2002). Common causes of work-related stress include work overload, time pressures, job insecurity, role conflict, organizational politics, difficult relationships, and lack of effective consultation (Cooper, 2013). As a result, 44% of workers worldwide (41% in the UK) report experiencing high stress on a daily basis (Gallup, 2022), impacting work performance and absenteeism (Park, 2007; Hassard et al., 2014), and potentially leading to burnout (Schaufeli et al., 2009). Work-related stress places significant costs on the economy through productivity losses (Hassard et al., 2018). A total of 17 million working days were lost due to work-related stress, depression, or anxiety in the UK in 2021/22 (Health and Safety Executive, 2022). The financial impact of the cost of work-related stress was previously estimated as £3.66bn in the UK (Health and Safety Executive, 2011).

Stress features in several organizational psychology theories. The Transactional Model of Stress and Coping (Lazarus and Folkman, 1984) shows how individuals appraise and experience stressors. The Job Demands-Resources (JD-R) model demonstrates how job demands (if not sufficiently moderated by job resources) lead to strain (Bakker and Demerouti, 2007). Stress and strain are sometimes used interchangeably, although strain is more accurately defined as “the individual’s psychological, physical, and behavioral responses to stressors” (Cooper et al., 2001, 14). Work stressors can be categorized as either challenges, that are supportive of personal growth, or hindrances, that are not (Cavanaugh et al., 2000). Within JD-R theory, challenges are positively associated with employee engagement, whereas hindrances are negatively associated (Crawford et al., 2010).

In terms of meaningful work, there is increasing interest from scholars of various disciplines, including positive psychology, organizational behavior, management studies and the humanities (Rosso et al., 2010). Unsurprisingly, the different disciplines bring different perspectives (Bailey et al., 2019b). From a positive psychology standpoint, meaningful work is a significant driver of meaning in life (Steger and Dik, 2009), which is a fundamental component of seminal theories of happiness like Psychological Wellbeing (Ryff, 1989) and PERMA (Seligman, 2012). Much of the management literature argues that organizations can (and should) encourage meaningfulness at work to support desirable organizational and individual outcomes (Fairlie, 2011; Michaelson et al., 2014). In contrast, within the humanities tradition, meaningfulness is an inherent quest and a basic human need (Frankl, 1964; Yeoman, 2014), that can’t easily be provided by an organization on behalf of its people (Bailey et al., 2017).

Despite—or perhaps because of—the increased interest in meaningful work (Steger, 2017), there remains considerable ambiguity about how it is defined, conceptualized, and operationalized (Both-Nwabuwe et al., 2017; Martela and Pessi, 2018). Indeed, Both-Nwabuwe et al. (2017) identified fourteen definitions each with corresponding different components, antecedents, and outcomes, and argued that this fragmentation is holding back the field. These different definitions draw on diverse perspectives, including that of the humanities tradition (in which the quest for meaningfulness is inherent), and that of positive psychology in which meaningful work is a multi-faceted eudaimonic state (Steger et al., 2013).

Linked to the different definitions, there are different ways of conceptualizing meaningful work. Some see meaningful work as unidimensional, combining various aspects of meaningfulness into a single experience (e.g., Carton, 2018; Allan et al., 2019). A more common view is that meaningful work is multidimensional (e.g., Rosso et al., 2010; Lips-Wiersma and Wright, 2012; Steger et al., 2012; Martela and Pessi, 2018). However, there is little consensus about the specifics of those dimensions. For example, Steger et al.’s (2012) three-dimensional model, consists of “experiencing positive meaning in work, sensing that work is a key avenue for making meaning, and perceiving one’s work to benefit some greater good” (p. 1). Lips-Wiersma and Wright (2012) conceptualize meaningful work along four dimensions: “developing the inner self, unity with others, service to others, and expressing full potential” (p. 660). With parallels to this, Rosso et al. (2010) see meaningful work as individuation, contribution, self-connection and unification, whilst Martela and Pessi (2018) conceptualize meaningful work as broader purpose and self-realization. There are also multiple operationalizations of meaningful work. The two most prominent validated measures are the Work and Meaning Inventory (WAMI; Steger et al., 2012) and the Comprehensive Meaningful Work Scale (CMWS; Lips-Wiersma and Wright, 2012). But there are also various other scales, and frequent use of bespoke measures (Both-Nwabuwe et al., 2017), making it harder to compare empirical research with confidence.

Adding to the complexity, meaningful work is closely linked to “neighboring concepts” (Martela and Pessi, 2018, 11), such as calling and workplace spirituality. Someone with a calling works for the fulfilment their job provides, rather than for salary or career progression (Wrzesniewski et al., 1997). Calling has similarities with meaningful work but is usually conceptualized as having a single overarching greater good (Steger et al., 2010)—for example, a feeling of “transcendent summons” (Dik and Duffy, 2009, 427)—whereas meaningful work is usually multi-dimensional (e.g., Lips-Wiersma and Wright, 2012; Steger et al., 2012). Workplace spirituality brings together meaningful work with a sense of spirituality and community to enhance organizational outcomes (Ashmos and Duchon, 2000).

Although there is no comprehensive theory of meaningful work (Lysova et al., 2019), the concept, like stress, features within several key organizational psychology theories. For example, in the Job Characteristics Model, meaningful work mediates job characteristics (skill variety, task identity, task significance, autonomy, and feedback) to create positive outcomes such as increased intrinsic motivation, improved performance, and increased job satisfaction (Hackman and Oldham, 1976). Within JD-R theory, meaningful work can be seen as a personal or psychological resource (Van den Heuvel et al., 2010). Like other resources, it counteracts the strain-inducing impact of job demands (such as mental, emotional, or physical challenges), leading to increased motivation and improved organizational outcomes (Bakker and Demerouti, 2007).

It is not just academia becoming fascinated by meaningful work. An increasing number of individuals are prepared to forego salary for meaningfulness (Ariely et al., 2008; Hu and Hirsh, 2017). As millennials become the dominant generation in the workplace, meaningfulness is becoming even more important (Ng et al., 2010). Responding to this, organizations are starting to ask how they can foster or facilitate meaningfulness for their employees (Dhingra et al., 2021; Eaton and Mallon, 2021). Lysova et al.’s (2019) multi-level review identified how meaningfulness can be fostered at four levels: individual, job, organization, and society. Although meaningful work is predominantly seen as a subjective and individual concept (Schnell et al., 2013), there are aspects that employers can influence (Cameron et al., 2003). Proactive steps which employers can take or facilitate include transformational leadership (Arnold et al., 2007), creating cultures that are innovative and supportive (Cardador and Rupp, 2011), and supporting individuals to engage in job crafting (Wrzesniewski and Dutton, 2001) and active use of their strengths (Littman-Ovadia and Steger, 2010).

Although empirical evidence remains sparse (Bailey et al., 2019b), that which exists details various wellbeing benefits of meaningful work, supporting the commonly held view of meaningfulness as being wholly positive. In a meta-analysis, Allan et al. (2019) found meaningful work has moderate to large correlations with meaning in life, life satisfaction and general health, and small to moderate negative correlations with negative affect. In addition, there is evidence of meaningful work being associated with reduced levels of stress and depression (Daniel, 2015), and improved work-to-life enrichment (Tummers and Knies, 2013; Johnson and Jiang, 2017). Self-oriented dimensions of meaningful work (such as integrity with self, expressing full potential) have stronger relationships with wellbeing than other-oriented dimensions (Lips-Wiersma et al., 2022), which the authors propose is due to other-oriented dimensions (such as service to others, balancing tensions) consuming more time and energy.

Meaningful work is also good for work performance and organizational outcomes. Allan et al.’s (2019) meta-analysis found large correlations between meaningful work and engagement, corroborating other studies not included within that meta-analysis (e.g., Albrecht, 2013; Fletcher et al., 2018). Moreover, meaningful work is the strongest predictor of engagement compared to other characteristics such as work relationships, intrinsic and extrinsic rewards, and leadership approach (Fairlie, 2011). Meaningful work is associated with increased job satisfaction (Duffy et al., 2011, 2013), organizational commitment (Duffy et al., 2011; Geldenhuys et al., 2014; Rawat and Nadavulakere, 2015), lower absenteeism (Steger et al., 2012; Soane et al., 2013) and lower intention to quit (Fairlie, 2011). Meaningful work is related to job performance, both self-reported (Pavlish and Hunt, 2012; Allan et al., 2017; Tong, 2018), and objective (Albuquerque et al., 2014). This is because meaningful work predicts engagement, commitment, and job satisfaction, which subsequently are associated with performance and organizational citizenship behaviors (Allan et al., 2019).

Although most empirical studies point to the benefits of meaningful work, there is a small and growing interest in possible negative outcomes (e.g., Mitra and Buzzanell, 2017; Magrizos et al., 2022). Seeking meaning from work can be harmful if pushed to excess (Bailey et al., 2019a). For example, a deep sense of calling was associated with foregoing pay and personal time (Bunderson and Thompson, 2009). Whilst the idea of a “golden mean,” derived from Aristotelian Virtue Ethics (Pakaluk, 2005), is not unusual, this is underexplored with regard to meaningfulness. There are unanswered questions about the optimal amount of meaningfulness at work, whether certain types of meaningfulness are more likely to predict harmful outcomes, and how meaningful work links to workaholism and other attributes (Bailey et al., 2019a). Indeed, some aspects of subjective meaningful work (specifically, service to others) are associated with resource loss, rather than resource gain (Lips-Wiersma et al., 2022). These negative outcomes have been characterized as a “painfully double-edged sword” (Bunderson and Thompson, 2009, 50), or the “dark side of meaningful work” (Duffy and Dik, 2013, 433), aligning with the movement in positive psychology to a wider consideration of the darker aspects of wellbeing (Lomas and Ivtzan, 2016). These downsides are under-researched (Bailey et al., 2019a) and yet to be incorporated into theoretical models.

Moreover, meaningful work can distract from and reduce engagement in other life domains, such as family time and self-care (Symon and Whiting, 2019), and it can be hard to prevent individuals from overworking when they find their work deeply meaningful (Mazzetti et al., 2014). Meaningful work is also associated with work devotion which can create conflict with loved ones through long and erratic working hours, and the erosion of work-life boundaries (Oelberger, 2019). When meaningfulness is challenged, people sometimes reframe their work in unhealthy ways to try to re-create the meaning they feel they have lost, leading to stress, anxiety and burnout (Florian et al., 2019). Academics who experience their work as a vocation experience simultaneous satisfaction and distress (Barcan, 2018).

The sense of meaningfulness can cause employees to accept challenging—even dangerous—working conditions, leading to personal sacrifices, exhaustion, and other negative health outcomes (Bergman Bruhn, 2022). Linked to this, in unscrupulous organizations meaningful work could be a mechanism to exploit employees (Bailey et al., 2017). Even well-intentioned organizations need to tread a fine line; attempts to foster meaningfulness for employees inauthentically may actually destroy meaning (Lips-Wiersma and Morris, 2009).

In terms of meaningful work and work-related stress specifically, the research is inconclusive, with no consensus on the nature of the relationships between the two. For example, a recent study by Lavy (2022) found a relationship between meaningfulness and increased stress in a sample of teachers. Other studies suggest more nuance, such as that of Lips-Wiersma et al. (2022) which found that expressing one’s full potential (as a component of meaningful work) is associated with higher stress, but other aspects (unity with others, service to others, and integrity with self) are associated with lower stress. Although meaningful work has been found to predict lower depression, it was only associated with lower stress when experienced alongside high job satisfaction (Allan et al., 2018). Daniel (2015) found a negative relationship between workplace spirituality (of which meaningfulness is a part) and stress, and several recent studies have shown that meaningfulness can help to reduce strain (Erlmaier et al., 2022; Gur et al., 2022; Mousa and Samara, 2022).

In summary, the complexities, tensions and “dark sides” (Bailey et al., 2019b) meaningful work remain underexplored, including what happens when meaningfulness becomes excessive (Johnson and Jiang, 2017), or how deeply meaningful work relates to common wellbeing risks such as work-related stress (Bailey et al., 2017; Oelberger, 2019). Empirical research is constrained by ambiguity in definition and the lack of consistent measurement scales and experimental studies. Some studies are focused on linked concepts such as workplace spirituality (e.g., Daniel, 2015), rather than meaningful work specifically. Moreover, as the vast majority of the research is from the USA (Allan et al., 2019; Bailey et al., 2019b), there is a danger of ethnocentrism and elitism in the meaningful work literature (Rosso et al., 2010). A comprehensive understanding is overdue: more individuals are wanting to experience meaning in their work (Hu and Hirsh, 2017), whilst work-related stress is widespread (Gallup, 2022) with significant health, work, and economic consequences (Michie, 2002; Cooper, 2013; Hassard et al., 2014).

If meaningful work is to be an asset for both individual wellbeing and organizational performance over the long-term, it is necessary to explore and appreciate its “intricate tensional knots” (Bailey et al., 2019a, 489). The aims of this research project are to understand the perspectives of people who find their work to be both deeply meaningful and stressful, and to explore the relationships between work-related meaningfulness and stress. A nuanced understanding would help people enjoy meaningful work as a positive force for wellbeing and protect against adverse consequences for individuals and their employers.

## Materials and methods

### Design

The study used a social constructivist grounded theory methodology. Grounded theory was selected given the absence of a comprehensive theory of meaningful work (Lysova et al., 2019). As an inductive approach, grounded theory is well suited to exploring complex, potentially contradictory situations (Edmondson and McManus, 2007). A social constructivist approach (Charmaz, 2006) was appropriate given the largely subjective nature of meaningful work (Schnell et al., 2013; Magrizos et al., 2022), and because many people construct meaning through conversations and interactions with other people (Florian et al., 2019; Martela et al., 2021). Constructivist grounded theory, as opposed to classic grounded theory (Glaser and Strauss, 1967) assumes that theories are constructed jointly by the participant and the researcher, rather than being entirely emergent. As researchers who have experienced work to be both deeply meaningful and at times very stressful, it was important to recognize and acknowledge our part in making meaning from the data, rather than standing apart from it. The study was reviewed and approved by the University of East London Ethics Committee.

### Participants

Seventeen (*N* = 17) participants were recruited for the study. All participants self-identified as finding their work meaningful and experiencing work-related stress in the last year. Participants were recruited through social media channels (primarily LinkedIn) and via the professional and personal networks of the first author. Participants provided written informed consent to participate in the study. The participants comprised 11 women (65%) and 6 men (35%), between the ages of 34 to 61 (*M* = 42.88, SD = 7.40). Participants were drawn from the private (47%), public (29%) and third (24%) sectors. Participant occupations included a healthcare assistant, General Practitioner (GP), architect and civil servant, plus those working at various levels in the education, energy, housing, and policing sectors, among others. All but one of the participants were based in the United Kingdom. Participants were not given any compensation or reward for their participation.

The study used maximum variation sampling to increase heterogeneity and potential generalizability. As part of this, mid-way though the recruitment period the advertisement was re-shared with particular encouragement for participants that were male, younger or working in the private sector. The advertisement was also shared in a Facebook group of over 20,000 members to counter potential bias from LinkedIn’s primarily professional users. Despite efforts to conduct purposeful sampling in this way, the study’s results are necessarily constrained by the networks that could be accessed within the timeframe.

### Materials

As a grounded theory study, data collection and analysis ran concurrently, with interview questions being iteratively informed by emerging findings from previous interviews (Foley et al., 2021). Questions were designed to be open, non-leading and non-judgmental, in order to best hear participants’ own perspectives (Charmaz, 2006). Some questions remained consistent throughout the interviews, for example, “What does meaningful work mean to you?” Other questions became more specific, for example, those relating to any potential relationships between meaningfulness and stress.

### Procedure and data analysis

Participants were invited to a semi-structured interview, held and recorded on Microsoft Teams and lasting around an hour. Interviews were transcribed by checking and amending the Teams transcript, using the video recording to clarify any key areas of emphasis, tone or body language. Participants were given pseudonyms, and identifying details such as the names of people, organizations, places, and job titles were redacted. Interviews were grouped into batches of three or four, enabling data analysis to commence in parallel with the collection of further data.

Data analysis commenced with initial coding (Charmaz, 2006), studying fragments of the text to understand their meaning and potential analytic value, supported by NVIVO software. In line with grounded theory’s inductive approach, the coding was led by the data, with no preconceived categories. Running data collection and analysis in parallel enabled the successive iteration of interview questions, and maximum variation sampling. Following initial coding, focused coding (Charmaz, 2006) was used to identify the categories that stood out as particularly interesting and prevalent; these were then tested against the rest of the data. Constant comparisons were made between data, categories, and cases throughout the analysis, seeking areas of similarity and difference (Glaser and Strauss, 1967). Other grounded theory coding techniques, such as axial coding and selective coding (Corbin and Strauss, 2008) were also explored. Although Charmaz (2006) doesn’t use these terms, they are consistent with the focused coding approach. Through axial coding it was possible to organize and re-organize the categories and sub-categories generated through the interviews, making sense of them and identifying connections. Selective coding was used to focus in on the key categories, which in turn shaped the final batch of four interviews to validate and challenge the emerging findings, with the aim of achieving data saturation. Finally, theoretical coding was used to identify the relationships between the key categories.

### Reflexivity

Throughout the data collection and data analysis stages, the first author wrote frequent memos (Charmaz, 2006), capturing thoughts, ideas and reflections on the data and what it might be saying. The subject of the memos varied considerably: some memos related to specific cases, others to categories or the relationship between categories, or to the author’s emerging thoughts about the limitations of the study.

## Results

The analysis produced an overarching theme that work-related meaningfulness and stress go “hand in hand” (in the words of a participant with the pseudonym Isabel). Collectively, the participants described several bi-directional relationships between meaningfulness and stress, experienced subjectively depending on the specific context and circumstances. Some relationships were perceived to be helpful to wellbeing, whilst others were seen to be harmful. The analysis identified six sub-themes below this overarching theme. Table 1 summarizes these sub-themes and details their prevalence within the data. Increasingly repetitive patterns were identified as the analysis progressed, particularly across the sub-themes with greatest prevalence (sub-themes 1, 2 and 3). The sub-themes will be discussed in turn, supported by quotes from participants (using pseudonyms).

**TABLE 1:** Summary of sub-themes, with their prevalence in the data.

Sub-theme		Prevalence
1	*Caring deeply*: meaningfulness can create or exacerbate stress	13/17
2	*A bottomless pit*: a high volume of meaningful work extends into other domains, increasing stress and reducing recovery time	11/17
3	*Stress alleviation:* meaningfulness can help to alleviate work-related stress	11/17
4	*Reinforcing meaningfulness:* Stress can reemphasize and reinforce the meaningfulness	7/17
5	*Reducing meaningfulness:* Stress can reduce and constrain the meaningfulness	6/17
6	*Inextricably linked:* Work meaningfulness and stress can be one and the same.	6/17

The inherent interconnectedness of meaningfulness and stress was summarized neatly by a participant who characterized it as two sides of the same coin. Patricia described a “virtuous circle” arising from her strong belief in the value of what she’s doing, and how that helps to alleviate some of the stress she experiences. Yet if the meaningfulness becomes challenged, leading to “stress for stress’s sake… then it becomes a very vicious circle.”

### *Sub-theme 1*. Caring deeply: meaningfulness can create or exacerbate stress (prevalence 13/17)

Participants reported that the meaningfulness they derive from their work leads them to care deeply about it, which in turn can create additional stressors or exacerbate existing stressors. As Karla, who works in a not-for-profit technology role, explained: “By definition, if something is meaningful for you, it means that you take care of it and it’s a priority, and you want to do it well… there will always be stress about performing well in something you care about.” Grace, a healthcare assistant, described it as having two sides: “A healthy side and an unhealthy side. The healthy side is absolute professional pride in wanting to deliver the very best care that you can. And the unhealthy side is doing that at almost any cost.” Emily, a civil servant, described how “you care more about the outcomes” when work is meaningful, but that this can be taken to extremes at times, devoting “almost too much love and focus.” The importance of the work was also emphasized by Barney, a GP, who said “because it’s meaningful it’s more stressful, because it matters… It adds a layer of stress because it matters.” Frank, in the housing sector, saw it as a linear relationship, in which “the more you care about it, there’s more potential for stress.”

Participants described how this deep passion and commitment led them to set and maintain high standards, sometimes excessively so. As Angela described, “it’d be dead easy to sit back and go, ‘Oh well, never mind’… But no, I want to deliver because I know the difference it’s going to make. I know how powerful it’s going to be.” For Patricia, a senior leader in higher education, caring greatly about the innovation inherent in her role created stress “pretty much every day, because we’re breaking so many boundaries. We’re doing so many things differently and it’s a meaningful decision to do things differently”. However, setbacks and delays can cause significant frustration and be taken to heart, as Angela went on to explain: “With the highs come the lows… and if you’re really passionate you tend to then get really, really frustrated.” Whilst for some people it might be socially desirable to be seen to work hard and care about your job, the participants’ stories appeared genuine and heartfelt. For example, Barney talked about how his motivation “to reduce the [health] inequality gap” was the “fundamental reason” why he was prepared to work “evenings, weekends, just constantly.” Emily described how her work-related stress was driven, in part because of “how important it felt like it was that we got it right.”

Participants also reported that caring deeply led them to worry about disappointing others: “The meaningfulness can cause stress for sure, because it can make you feel a much greater need to succeed, and not let people down” (Emily). Other implications of caring deeply leading to higher stress include “sleepless nights” (Ollie), “being subsumed by my job” (Patricia), occasions when “it boiled over too much” (Hamish), and “los[ing] the balance and perspective” (Emily).

### *Sub-theme 2*. A bottomless pit: a high volume of meaningful work bleeds into other domains, increasing stress and reducing recovery time (prevalence 11/17)

Most participants described how there is always more meaningful work to do. Barney described his work as “a bottomless pit” and said, “you could just work 24 hours a day and never sleep.” The volume of work demands a high level of discretionary effort, far exceeding participants’ contracted hours. Participants described regularly “putting in a huge amount of extra hours” (Frank), and feeling compelled to do so, such as Chandni who said “often you don’t leave till 7 or 8 p.m. Because you can’t. Because you can’t go… There’s things to do, things to be done.” Similarly, Grace reported “feeling like you need to cover shifts on top of your hours because [other] people aren’t there.” However, the meaningfulness of the work means that participants are less likely to limit or withdraw their discretionary effort, even in the face of significant challenges. As a result, the volume of meaningful work seems to further compound work-related stressors.

Participants were emotionally connected with the positive (sometimes transformational) impact for the people who benefit from their work, which can make it harder to determine what’s sufficient and when to draw the line. Angela described the challenge of determining “enough” in her leadership role: “I really beat myself up about a lack of progress… I always think I haven’t done enough.” Similarly, Emily noted the judgments involved with determining when to stop: “There’s a degree of how well you can do it. There’s a definite spectrum: you can do it to the minimum, or you can go a lot further.” Barney echoed this: “the fact that it’s meaningful and a bottomless pit makes it much more difficult to get the balance right.” Other participants described the challenge of switching off from work, particularly in technology-enabled “always on” work cultures: “the grind of…. ping, ping, ping” from email notifications (Madeleine) and “checking my phone a lot, late into the evening and at the weekends” (Emily). For some, it was less the volume of work, and more the fear of missing out: “I just don’t want to miss anything, because policing is 24/7” (Leena).

As a result of the volume of deeply meaningful work, participants have reduced time for other aspects of their life. Some reported family challenges such as “not seeing my wife, not seeing the children” (Barney), due to excessive working hours including evenings and weekends. Others have had to stand down from community responsibilities, such as Madeleine: “I was on the church cleaning rota. And I was like, I can’t even clean my own home, let alone clean the church, you know?” For others, a high volume of meaningful work makes it harder to recover from work pressures, and be in the moment during non-work time: “It [work] does tend to permeate… I do think about it quite a lot” (Jillian); “I don’t have enough energy for the other things that are important… my husband and friends and family” (Grace); or “with my kids, for example, I was never fully present … you’ve always half a mind on all the things you’ve got to do” (Emily).

### *Sub-theme 3*. Stress alleviation: Meaningfulness can help to alleviate work-related stress (prevalence 11/17)

Participants reported that experiencing their work as meaningful helped them to tolerate and manage work-related stress. Generally, this seemed to be because they accept that the stress is in service of the important outcomes of their work, which they prize deeply. Patricia described how the meaningfulness enables her to see the stress as a price worth paying: “I can cope with a lot more stress if I can see that I’m adding value, whereas if it’s just stress for the sake of stress then I find it a lot harder to deal with.” Similarly, for Hamish: “I think it’s less stressful when there’s a positive impact of what you’re doing.” Nicholas, a counselor, likened experiencing extreme meaningfulness and stress to soldiers fighting a war: “Fighting a war is fine if you believe it’s a just war. But thinking you might kill or give your life for a war that you don’t think is just, that’s appalling.” This example also emphasizes the subjective nature of meaningfulness, which was implied in most participants’ responses.

As well as significant job satisfaction, participants reported that meaningfulness helped them “be able to get up and go to work every day” (Danielle), “persevere” (Ollie) and stay motivated, such as Chandni who shared that “it energizes you to keep giving your best.” Linked to the previous sub-theme, meaningfulness also enabled participants to “justify working a bit harder” (Hamish), despite the presence of stressors. The stress-alleviating properties of meaningful work can enable people to tolerate difficult circumstances, highlighting meaning’s usefulness as a coping strategy. Grace described how the meaningfulness “makes even the very, very hard days worthwhile,” and Frank shared how “it’s easier to get out of those ruts when you know you’re doing something to try and help someone have a better life.”

Meaningfulness can also help people stay in a role despite challenging, even toxic, circumstances. Leena described how she has “thought about moving on… and thought no, I’ll work out how I can make this work, but I’m not leaving this organisation at the end of the day.” Whilst it’s possible Leena was using meaningfulness as a reason to avoid the upheaval of looking for a new role, the importance she saw in the “bigger picture… what we’re doing impacting the public” suggested her experiences of meaning gave her sufficient reason to stay. Isabel felt similar: “I would just quit if it wasn’t for the fact that you do get this buzz when you achieve something.”

Whilst the COVID-19 pandemic created additional stressors for many participants, for Barney it also meant a significant boost in meaningfulness which more than offset the stress: “Even though I worked incredibly hard for that finite time period, I didn’t feel particularly stressed or burned out, because I knew what I was doing was so important and meaningful.”

### *Sub-theme 4*. Reinforcing meaning: Stress can reemphasize and reinforce the meaningfulness (prevalence 7/17)

For some participants, work stress can bring meaningfulness into sharper focus. As Grace described: “When things are particularly hard, you’re reminded why you’re doing it.” Participants reported that the challenges they faced made their work feel more tangible and reemphasized its value, for example Leena, reflecting on the most difficult aspects of her role: “It’s not really a negative. I think it just makes it more real. It makes me realize how important the role is, even more so.” Emily shared how stressful circumstances (in this case helping to deliver a high-profile national event) can reinforce meaningfulness and create positive emotion:

Obviously, there’s a sense of stress. We’ve got to do a good job here and the expectation is huge. But it does also remind you of the—oh my god, this is quite a historic thing we’re working on. The stress and buzz and excitement.

Ollie experienced a reinforcement too: “I suppose in my brain there’s a positive reinforcement thing that anything I’m stressing about is super-important… so it probably does reinforce that meaningfulness side of things.” For Isabel, who works in adult social care, this relationship felt linear: “The more challenges you face with a client, the more meaningful it is when it’s done.”

### *Sub-theme 5*. Reducing meaning: Stress can reduce and constrain the meaningfulness (prevalence 6/17)

For some participants, the stress can lead to a reduction in meaningfulness. These participants reported feeling less engaged due to the stressors, as described by Emily: “You still feel like it’s important, but it’s like… why are you bothering with it? Because it feels like you’re not getting on or making progress. And I think that can be quite hard work.” Participants experiencing stress related to job insecurity described how this contributed to reduced meaningfulness. Jillian explained: “The stress of not knowing whether myself or my team was going to have a job…. That takes a bit of the meaning out of your job.” Whilst for Karla, it was stressors related to poorly managed organizational change that reduced her sense of meaning: “I think when I was most disillusioned, when I really questioned my place, was when my team just got moved from one place to another without being consulted… Why didn’t I know? Why did nobody talk to me?” For some, reduced meaningfulness caused by stress prompted them to consider finding a new role, such as Madeleine: “I suppose when I was really ready to resign and say that’s it, that would have been a reaction to it just all being too much. And it was because I’m just not finding any meaning.”

### *Sub-theme 6*. Inextricable: Work meaningfulness and stress can be one and the same (prevalence 6/17)

As shown in the sub-theme prevalence, most participants identified multiple relationships between work meaningfulness and stress. Some of these relationships were seen by participants to be positive for wellbeing; specifically, sub-themes 3 (meaningfulness alleviates stress) and 4 (stress reinforces meaningfulness). Other relationships were seen as being deleterious; specifically, sub-themes 1 (caring deeply, leading to stress), 2 (a bottomless pit of meaningful work), and 5 (stress reduces meaningfulness).

In most cases, participants experienced both helpful and unhelpful relationships between meaningfulness and stress, sometimes at the same time. For example, nine participants reported that caring deeply about their work created or exacerbated stress (sub-theme 1) and that meaningfulness helped them to alleviate their stress (sub-theme 3). Ollie described this duality by saying: “Sometimes work is incredibly difficult and horrible and frustrating. But because we’re doing some good, I do it. And I enjoy doing it.” Not only were meaningfulness and stress described as going “hand in hand” (Isabel), but also more proactively “egg[ing] each other on” (Hamish) which implies mutual reinforcement via a positive feedback loop.

Similarly, three participants reported that stress can both reinforce meaningfulness (sub-theme 4) and reduce meaningfulness (sub-theme 5). On this point, Grace made a distinction between different types of stressors, and how they might affect meaningfulness differently:

For me personally, I think the quantity stresses [stressors related to the volume of work] do reduce the meaningfulness a bit, and I could see that if those were dialed up that your meaningfulness would end up getting elided overtime. But [stressors related to] the complexity probably has the opposite effect… having those difficult moments does remind you of why you’re doing what you’re doing. So I think it depends on what type of stress.

Some participants saw the bi-directional relationships between meaningfulness and stress as not just ambivalent (both helpful and unhelpful), but also as inextricably linked, such that one can’t exist without the other. Nicholas summarized it with a phrase his late father had regularly used: “few things in life are easy and worth doing.” Without stress there can be no meaningfulness, and perhaps vice versa.

Angela described the role of stress in creating meaning and personal accomplishment, saying “I feel like you need to go through those conflicts to feel like you’ve delivered something meaningful… Look what I achieved, despite all the odds.” This was reiterated by Isabel who emphasized “if it wasn’t stressful it wouldn’t be meaningful to me…. It’s that challenge, it’s that buzz, it’s that rush that’s caused by the stress… If it wasn’t for that, you wouldn’t get the meaningfulness.” Not only is this duality inherent, but it would also be some participants’ active choice: “I don’t want it to become non-meaningful but easy” (Chandni).

## Discussion

**FIGURE 1:**
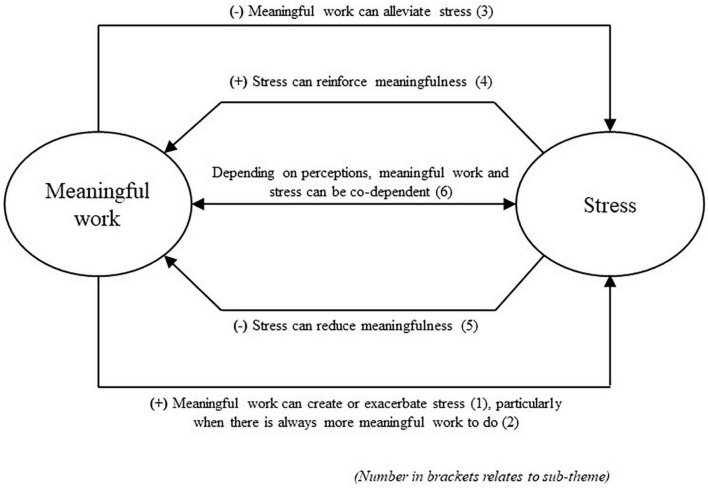
Model depicting the six sub-themes.

The study aimed to understand the perspectives of people who find their work both meaningful and stressful, and to explore the relationships between the meaningfulness and the stress. In so doing, it intended to help understand how meaningful work can be enjoyed as a positive force for wellbeing, avoiding adverse consequences for individuals and their employers. Each sub-theme is depicted in the model at Figure 1. This simple model demonstrates the multiple relationships between the two concepts, how these relationships can create both helpful and unhelpful outcomes, and the potential for meaningfulness and stress to be mutually dependent. The results suggest that meaningful work and work-related stress are inherently connected, with multiple bi-directional relationships that both support and hinder wellbeing. Specifically, it is beneficial that meaningfulness can alleviate stress (sub-theme 3), and that stress can re-emphasize meaningfulness (sub-theme 4). Yet it is detrimental that meaningfulness can create or exacerbate stress (sub-theme 1), particularly when the volume of work is significant (sub-theme 2), and that stress can reduce meaningfulness in some circumstances (sub-theme 5). A final sub-theme goes further than the overarching theme of multiple bi-directional relationships; depending on individuals’ perceptions of meaningfulness and stress, the two might be seen as going “hand in hand.” That is, you can’t have one without the other (sub-theme 6). Participants described the competing tensions that can create the bi-directionality in the relationships between meaningfulness and stress. For example, feeling a significant sense of purpose and mission in work can lead someone to take on additional responsibilities or push for higher standards, creating additional stress (sub-theme 1). This can also be exacerbated by taking frustrations and setbacks personally, because they feel such a strong emotional attachment to their work. This increased pressure can either accentuate the meaningfulness they feel in their work, encouraging them to put in further discretionary effort (sub-theme 4), or, it can reduce their sense of meaningfulness, which—if persistent and unchecked—can cause them to become disillusioned with their work (sub-theme 5).

Overall, these results challenge the view that meaningfulness is always a good thing (Michaelson et al., 2014). Instead, the results provide further support for meaningful work as ambivalent (Magrizos et al., 2022) and potentially paradoxical (Bailey et al., 2019a), showing that meaningfulness can be both helpful and unhelpful, depending on the circumstances and individual perception. Whilst the finding that meaningfulness can help alleviate stress (sub-theme 3) supports the evidence for the wellbeing benefits of meaningfulness (Allan et al., 2019; Bailey et al., 2019b), the finding that meaningfulness can exacerbate or create additional stress (sub-theme 1) indicates that the relationship is more complex. Overall, the results endorse Mitra and Buzzanell (2017) tensional approach and emphasis on the dualities of organizational life, in which they describe meaningfulness as “a dynamic and contested negotiation, rather than a purely positive outcome” (p. 595). As expected, participants’ interpretations of meaningfulness were personal to their circumstances and experiences, supporting the predominant view of meaningful work as a subjective concept (Schnell et al., 2013). Several participants reflected on how the interview helped them to re-connect with their sense of meaningfulness, validating the use of a constructivist approach to the research.

### Caring deeply: meaningfulness can create or exacerbate stress

This finding supports previous studies that show how meaningful work leads to a deeper sense of commitment, even a sense of duty, which can magnify and exacerbate stressors (Bunderson and Thompson, 2009; Bergman Bruhn, 2022). A recent study (Lavy, 2022) found that teachers’ daily sense of meaningfulness was associated with increased stress the following day, which could be due to an increased sense of responsibility to others. This echoes findings (Lips-Wiersma et al., 2022) that service to others is associated with resource loss. Caring deeply, with its connotations of emotional connection, dedication and even love, also shares similarities with work devotion, which is associated with meaningful work and could lead to self-sacrificing behaviors and conflict with loved ones (Oelberger, 2019).

The finding also suggests that it’s possible for meaningfulness to become excessive (Johnson and Jiang, 2017). This could imply that there’s an optimum amount of meaning, echoing Aristotle’s golden mean (Pakaluk, 2005). However, this may be overly simplistic: bi-directional relationships that are both positive and negative, sometimes concurrently, are evident in the data. Moreover, as perceptions of meaningfulness are largely subjective and context-dependent, it is unlikely there is a single “optimum” level of meaningfulness, even at an individual level.

### A bottomless pit: a high volume of meaningful work extends into other domains, increasing stress and reducing recovery time

Where participants experienced their work to be both meaningful and seemingly unending, it was even more likely to encroach into their personal life through long hours and finding it hard to switch off (Mazzetti et al., 2014; Symon and Whiting, 2019). Magrizos et al. (2022) found that people with low workaholism experienced a positive and linear relationship between meaningful work and work stress, whereas those with high workaholism experienced a non-linear relationship. In effect, workaholics who experienced significant meaning also experienced significant stress as well. Although workaholism wasn’t explored specifically in the study, one participant alluded to it when explaining how she liked to be “subsumed” by her work (Patricia), and others such as Angela talked about “passion” for their work. Indeed, the findings may echo aspects of Vallerand et al. (2003) dualistic model of passion, in which passion can be either harmonious or obsessive. Harmonious passion is pursued with balance to other domains and linked with positive affect, but obsessive passion is pursued in conflict with other domains, causing negative affect and problems in other areas of life.

### Stress alleviation: meaningfulness can also help to alleviate work-related stress

This finding aligns with other studies which have found that meaningfulness can help to reduce strain (Erlmaier et al., 2022; Gur et al., 2022; Mousa and Samara, 2022). More specifically, meaningful work significantly buffers hindrance stressors (job demands that don’t support personal growth, such as office politics), but not challenge stressors (job demands that do support growth, such as high responsibility) (Meng et al., 2022). This might account, in part at least, for some of the ambivalence and how sub-themes 1 and 3 might appear to contradict each other.

### Reinforcing meaningfulness: stress can reemphasize and reinforce the meaningfulness

Some participants reported that stress reinforced their sense of meaningfulness. Our interpretation is that the stressors highlight the motivational aspects of why they care about their work, increasing the salience of the consequences and sense of meaningfulness. However, it might also be possible that the stressors actually challenged their sense of meaning, causing them to cognitively re-frame how they perceive the work in order to not “lose” meaningfulness (Florian et al., 2019). If so, this sub-theme (whilst appearing positive) could actually lead to increased stress (as Florian et al., 2019) and become a vicious cycle.

### Reducing meaningfulness: stress can reduce and constrain the meaningfulness

Erlmaier et al. (2022) found that meaningfulness can alleviate work strain (sub-theme 3) but also found evidence of a bi-directional relationship, in that experiencing work strain reduced perceptions of meaningfulness, which is supported by this finding (sub-theme 5). The apparent contradiction between sub-themes 4 and 5 might be partially explained by individual differences: some people reported that stress reinforced meaningfulness, and others found that stress reduced meaningfulness. However, as noted previously, three participants experienced both reinforcement and reduction of meaningfulness. This suggests that whilst individual differences may play a role, for some people the specific nature of the stress or wider circumstances may also have an impact. For example, perhaps meaningfulness responds differently to stressors perceived to be hindrances rather than challenges (Cavanaugh et al., 2000).

### Inextricably linked: work meaningfulness and stress can be one and the same

This finding provides support for the poignancy of meaningful work, characterized as much by discomfort and challenges as by satisfaction and successes (Bailey and Madden, 2016). It also supports the fundamental tension identified by Bunderson and Thompson (2009) that “deep meaning does not come without real responsibility” (p. 52). Perhaps meaningfulness and stress are actually one and the same: for something to be meaningful it means it matters and is important and is therefore more likely to create anxieties and stress.

Indeed, the experience of meaningfulness might be dialectical, that is, relating to the interaction between opposing forces. Lomas and Ivtzan (2016) described the principle of co-valence with regard to emotional states, and how they can be “complex, intertwined shared of light and dark” (p. 1755). Whilst not an emotional state, these results suggest that meaningfulness might be described as co-valent or ambivalent due to the coexistence of seemingly opposing characteristics. These results beg the question: are the downsides of meaningfulness (in this case, stress) an inherent and essential part of the experience?

### Findings in relation to the job demands-resources model

In the absence of a comprehensive theory of meaningful work (Lysova et al., 2019), Figure 2 shows how these results might be understood in the context of the JD-R model (Bakker and Demerouti, 2007). Within JD-R theory, meaningfulness is usually seen as either a personal resource that acts similar to a job resource (Van den Heuvel et al., 2010), or a psychological state created by resources (Albrecht et al., 2021). Stressors are job demands that can lead to strain. Some findings fully support the model (specifically, sub-themes 2, 3, and 5). Other findings (sub-themes 1, 4, and 6) challenge it and add weight to calls for a more nuanced understanding of how personal resources operate within job demands-resources theory (Schaufeli and Taris, 2014):

• Sub-theme 1: Despite being a resource, meaningfulness can actually create or exacerbate strain. This finding challenges the usual interpretation of the JD-R model, which holds that resources buffer demands. It might be explained by the ambivalence of meaningful work, for example leading to a curvilinear relationship whereby meaningfulness is only helpful up to a certain point, and then becomes unhelpful.
• Sub-theme 2: A significantly high volume of meaningful work increases job demands, leading to additional strain; this aligns to the effect of other job demands.
• Sub-theme 3: Meaningful work can help to alleviate stressors; this aligns to other resources buffering the impact of demands on strain.
• Sub-theme 4: Stressors can reinforce the meaningfulness. This finding challenges the usual interpretation of the JD-R model, which holds that demands reduce job resources and motivation. It might be explained by participants subconsciously using cognitive re-framing to retain their sense of meaningfulness (Florian et al., 2019). Alternatively, it may be that challenges (stressors that support personal growth) and hindrances (stressors that do not support growth) are responding in different ways (Cavanaugh et al., 2000), but this is masked within the data.
• Sub-theme 5: Stressors can reduce the meaningfulness, in a similar manner to other job demands.
• Sub-theme 6: Meaningfulness and stress are inherently connected, and indeed co-dependent to an extent, as summarized in Nicholas’s phrase: “few things in life are easy and worth doing.” This finding challenges the usual interpretation of the JD-R model, which does not see demands and resources as co-dependent. It may be that meaningful work is an outlier amid other resources, however, this raises questions about whether other personal resources (e.g., self-efficacy) might work in similar ways.

**FIGURE 2:**
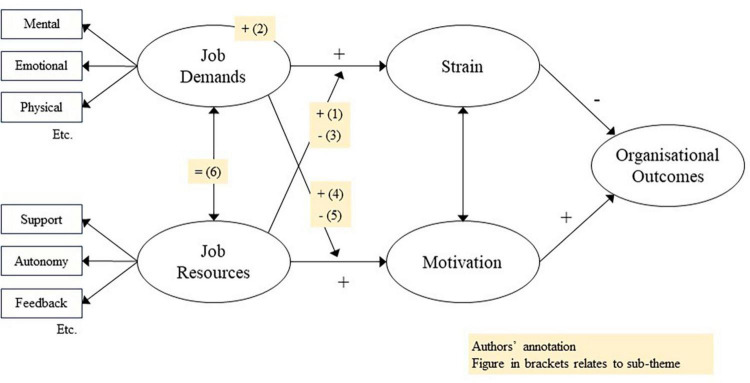
Job demands-resource model, annotated to represent this study’s findings.

The Job Demands-Resources model is a well-established and flexible model of the motivation and strain processes within work. However, as a descriptive (rather than explanatory) model, there remain significant unanswered questions about why particular demands and resources interact as they do (Schaufeli and Taris, 2014). These findings add to that list of unresolved issues, raising the prospect of different impacts on engagement and strain depending on personal perceptions of demands and resources, particularly subjective resources such as meaningfulness.

### Limitations of the current study

There are several limitations of this study. As a social constructivist study, the authors acknowledge the biases brought to the project and how these can impact sampling, data collection and analysis. The study purposefully sought participants who find their work both meaningful and stressful. It is inevitable that the findings would differ if participants who found their work meaningful but not stressful were included, should such a population exist. Whilst the study’s findings remain of significant interest given the increasing appeal of meaningful work (Hu and Hirsh, 2017) and prevalence of stress (Gallup, 2022), a larger representative sample would be required to examine whether stress and meaningfulness tend to coexist in the general population. Unfortunately, due to the constraints on timescales for the project, saturation was not achieved. Saturation is notably difficult to achieve within time-limited projects (Timonen et al., 2018), although the sample (*N* = 17) was close to Creswell’s (2009) advice that 20 participants are sufficient for a grounded theory study. Increasingly repetitive patterns were identified in later analysis, particularly across the sub-themes with greatest prevalence (sub-themes 1, 2, and 3), so the impact of meaningfulness on stress was well expressed, though participants may have responded to demand characteristics driven by the recruitment criteria, possibly exaggerating their experiences of meaningfulness or stress, or overstating the relationships they perceive between the two. Some people believe it is socially desirable to have meaningful and stressful work, perhaps as part of a cult of “busyness” (Bellezza et al., 2017), and this may have influenced data collection.

Another potential sampling bias arises from the use of the first author’s networks for participant recruitment. This risked attracting people that share similar backgrounds and beliefs, despite purposeful sampling efforts to attract a reasonable level of demographic and sector diversity. The study provides a monocultural perspective, with all but one of the participants based in the UK. The Protestant work ethic (Van Hoorn and Maseland, 2013) may play a part in sub-themes 1 and 2, in terms of participants’ commitment to their work, and propensity to work hard for something they care about, and thus these findings may not hold cross-culturally. Recent research has suggested a mediating role of family work conflict in decreasing a sense of meaningful work (Vara-Horna and Espinosa-Domínguez, 2023), but no questions on family or home life were included in this study. Future research could deliberately seek to cross-culturally validate these findings with a more diverse sample, and specifically inquire about the impact on, and impact of, family life.

Regarding potential data collection and analysis biases, questions were iterated by the first author; it was beyond the scope of the study to have a second researcher to support the data collection. It is possible that this may have introduced bias, wherein codes of particular interest to the researcher were prioritized, despite the considerable efforts to use open questions and be reflexive (including through memo-writing, and by the authors actively identifying and challenging assumptions and testing key concepts with others). Moreover, interpretations have been constructed from participants’ responses through the lens of the authors’ perceptions, which are, by definition, subjective rather than objective. The interconnectedness of the sub-themes makes it challenging to speculate how these possible biases may have affected specific individual themes. It is possible that neoliberal cultural ideals may mediate the very relationship between meaningfulness and stress, as the cult of “busyness” (Bellezza et al., 2017) creates a social stigma around lack of stress and implies anything meaningful is also stressful. Whilst this could be limited to Protestant cultures, this relationship may simply be tautological from the definitions of the words, as meaning and stress both imply some positive impact if successful and cost of failure if not. Are meaning and stress two aspects of the same experience?

The study has other limitations, including those often leveled at qualitative studies such as a lack of reproducibility (mitigated by articulating the method and through interview protocols) and lack of generalizability (managed through purposeful sampling). It is also cross-sectional, so doesn’t help explore how the relationships between meaningfulness and stress might change over time. For example, Bergman Bruhn (2022) found that meaningfulness buffered strain from substandard working conditions in the short-term but appeared to contribute to strain over the long-term. Finally, as a solely qualitative study it was not possible to measure participants’ level of meaningfulness or stress (to the extent possible, given the subjective nature of both concepts).

### Contribution to the field, and implications for research, policy and practice

This study indicates that meaningful work and work-related stress are inherently connected, and that the relationships between the two can both support and hinder wellbeing. It raises further questions that can’t easily be explained by existing theories such as the Job Demands-Resources model, such as in what circumstances might meaningfulness become detrimental, and why some stressors can feel supportive of meaningfulness.

There appear to be directional and causal relationships between meaningful work and work-related stress which could be validated or challenged through quantitative studies. Disentangling the proposed relationships detailed in Figure 1 is a complicated process. Meaningfulness appears to have both positive and negative impacts on stress and vice versa, so measures and models would need to consider the conflicting nature of this relationship. Any simple cross-sectional design using only single measures of stress and meaning would be inadequate for differentiating the positive and negative pathways between the variables. Instead, future studies should seek to differentiate between two types or aspects of meaningfulness: that which increases stress and that which reduces stress. Similar studies could be conducted on stress, attempting to identify the latent variables that power the effect of each (positive and negative) pathway. Structural Equation Modeling could be a suitable method to infer the latent variables that cause each effect and conduct a path analysis. Understanding the circumstances (context, types of stressors, individual differences) under which work stress might reinforce (rather than reduce) meaningfulness, and the impact of individual perceptions of both stress and meaningfulness would inform a refreshed and more nuanced understanding of the JD-R model, with greater distinction between objective and subjective assessments of both demands and resources. Furthermore, they may contribute to the development of a comprehensive theory of meaningful work to the benefit of employees, employers, and the economy.

Future research could also work to address the other limitations of this study. For example, qualitative studies could continue the interviews to ensure data saturation and conduct data analysis with multiple coders to reduce bias and incorporate more diverse perspectives. Future studies could explore meaningfulness and stress in other cultures, particularly those with less Protestant influence, and also between cultures, for example, comparing experiences of employees within a multinational company. Longitudinal studies could explore how the relationships between meaningful work and stress change over time and in response to world events. Studies could take into account demographic differences such as gender, age, ethnic diversity, marital status or family circumstances. Future studies could also employ a mixed methods approach, perhaps using a meaningful work scale (e.g., Steger et al., 2012) and a stress scale (e.g., Cohen et al., 1983) to measure participants’ self-reported meaningfulness and stress, before using qualitative methods to explore these experiences in detail.

Finally, these results demonstrate the duality and complexity of meaningful work with regard only to stress and might raise similar questions for the relationships between meaningfulness and other work-related outcomes. Meaningfulness has been shown to be associated with improved work outcomes such as engagement (Allan et al., 2019), job satisfaction (Duffy et al., 2011), and job performance (Allan et al., 2017). Given the links between stress and these outcomes, further research may help identify whether these relationships are straight-forward or, as these findings suggest is the case for wellbeing, more complex.

In terms of the implications for policy and practice, this study provides support for a more nuanced discussion around meaningful work. With more people seeking meaningfulness from their work (Hu and Hirsh, 2017) and various interventions seeking to help create that meaning (e.g., Wrzesniewski and Dutton, 2001), there is a risk that meaningful work is seen as a “silver bullet” to cure all ills. These results show that there are both upsides and downsides to meaningful work. By acknowledging and discussing the downsides in wellbeing seminars and coaching sessions, for example, it will help individuals be alive to potential risks, and better tread the line between meaningfulness and stress.

In a similar vein, organizations should also be mindful of the potential dark sides of meaningfulness for their people. That is not to say that organizations shouldn’t still promote the virtues of meaningfulness or help facilitate it for their staff, as proposed by Fairlie (2011), Pratt et al. (2013), and Michaelson et al. (2014). But efforts to do so should include discussion about the potential downsides and how, in some circumstances, meaningfulness can also have implications for stress.

## Conclusion

Through a social constructivist lens, the study has explored participants’ perspectives and perceived realities of meaningful work and work-related stress. The results suggest the presence of multiple bi-directional relationships between meaningful work and work-related stress, which can be both helpful and harmful for wellbeing. The study also proposes that meaningfulness and stress might even be inherently co-dependent for some people, such that they can’t experience one without the other. This supports the view of meaningful work as being complex (Mitra and Buzzanell, 2017), and no panacea for wellbeing. Instead, the situation is more complex, and reflects the dualities of modern life and work. Considering meaningful work holistically, both light and dark sides together, will be more beneficial for individuals and their employers. This may require further resources and more nuanced analysis, but few things in life are easy *and* worth doing.

## Data availability statement

The raw data supporting the conclusions of this article will be made available by the authors, without undue reservation.

## Ethics statement

The studies involving humans were approved by the University of East London Psychology Ethics Committee. The studies were conducted in accordance with the local legislation and institutional requirements. The participants provided their written informed consent to participate in this study.

## Author contributions

JA collated the data collection and led the analysis, and wrote the first draft of the manuscript. Both authors contributed to conception and design of the study, manuscript revision, read, and approved the submitted version.
